# A target containing phantom for accuracy assessment of cone‐beam CT‐guided histotripsy

**DOI:** 10.1002/acm2.14329

**Published:** 2024-03-18

**Authors:** Ayca Z. Kutlu, Grace M. Minesinger, Paul F. Laeseke, Michael Speidel, Martin G. Wagner

**Affiliations:** ^1^ Department of Radiology University of Wisconsin‐Madison Madison Wisconsin USA; ^2^ Department of Medical Physics University of Wisconsin‐Madison Madison Wisconsin USA; ^3^ Department of Biomedical Engineering University of Wisconsin‐Madison Madison Wisconsin USA; ^4^ Department of Medicine University of Wisconsin‐Madison Madison Wisconsin USA

**Keywords:** accuracy improvement, cone beam CT, histotripsy, phantom, quality assurance, targeting

## Abstract

**Purpose:**

Histotripsy is a nonionizing, noninvasive, and nonthermal focal tumor therapy. Cone‐beam computed tomography (CBCT) guidance was developed for targeting tumors not visible on ultrasound. This approach assumes cavitation is formed at the geometrical focal point of the therapy transducer. In practice, the exact location might vary slightly between transducers. In this study, we present a phantom with an embedded target to evaluate CBCT‐guided histotripsy accuracy and assess the completeness of treatments.

**Methods:**

Spherical (2.8 cm) targets with alternating layers of agar and radiopaque barium were embedded in larger phantoms with similar layers. The layer geometry was designed so that targets were visible on pre‐treatment CBCT scans. The actual histotripsy treatment zone was visualized via the mixing of adjacent barium and agar layers in post‐treatment CBCT images. CBCT‐guided histotripsy treatments of the targets were performed in six phantoms. Offsets between planned and actual treatment zones were measured and used for calibration refinement. To measure targeting accuracy after calibration refinement, six additional phantoms were treated. In a separate investigation, two groups (*N* = 3) of phantoms were treated to assess visualization of incomplete treatments (“undertreatment” group: 2 cm treatment within 2.8 cm tumor, “mistarget” group: 2.8 cm treatment intentionally shifted laterally). Treatment zones were segmented (3D Slicer 5.0.3), and the centroid distance between the prescribed target and actual treatment zones was quantified.

**Results:**

In the calibration refinement group, a 2 mm offset in the direction of ultrasound propagation (Z) was measured. After calibration refinement, the centroid‐to‐centroid distance between prescribed and actual treatment volumes was 0.5 ± 0.2 mm. Average difference between the prescribed and measured treatment sizes in the incomplete treatment groups was 0.5 ± 0.7 mm. In the mistarget group, the distance between prescribed and measured shifts was 0.2 ± 0.1 mm.

**Conclusion:**

The proposed prototype phantom allowed for accurate measurement of treatment size and location, and the CBCT visible target provided a simple way to detect misalignments for preliminary quality assurance of CBCT‐guided histotripsy.

## 1 INTRODUCTION

Histotripsy is a nonionizing, noninvasive, and nonthermal tissue focal tumor treatment that creates mechanical destruction via short duration (< 20 µs), high‐pressure (> 15 MPa) focused ultrasound pulses.^1^ Multi‐element curvilinear histotripsy therapy transducers transmit ultrasound pulses into target tissue through a coupling medium (degassed water).^2^ Cavitation is the rapid expansion and collapse of tissue intrinsic gas microbubbles under high negative pressures, a threshold phenomenon that occurs at the therapeutic ultrasound beam focus.^3^ Cellular structures turn into an acellular homogenate, as they are mechanically sheared within cavitation bubble clouds.^4^

Previous studies have demonstrated the distinct tissue effects of histotripsy compared to other cancer therapies.^5^ The susceptibility of tissues to histotripsy is dependent on tissue collagen content.^5^, ^6^ Tissue selectivity can allow for safe parenchymal tissue destruction and preservation of vital fibrinous tissue including vessels and bile ducts within treatment zones.^7^ A multi‐center phase 1 clinical trial has demonstrated the safety and efficacy of in‐human histotripsy as a novel liver cancer therapy.^8^ The unique abscopal effects of histotripsy previously demonstrated in animal studies were also observed in humans in the trial.^8^, ^9^, ^10^

Histotripsy is currently guided by diagnostic ultrasound. Large body habitus, gastrointestinal gas, and bone in the diagnostic beam path can obscure the visualization of a tumor and prohibit targeting. To increase the number of tumors treatable by histotripsy, cone‐beam computed tomography (CBCT) targeting was developed, where a robotic arm is used to position the therapeutic transducer based on CBCT imaging.^11^, ^12^ The robotic arm is registered to the CBCT coordinate system such that the focal point of the transducer can be automatically aligned on a target selected in a planning CBCT of the patient. Treatment volumes are then created by an automated robotic arm joint movement of the therapy transducer through a planned volume of any shape and size.^7^, ^13^, ^14^

The CBCT targeting approach assumes the cavitation bubble cloud forms at a fixed location relative to the tip of the robotic arm corresponding to the geometrical focal point of a multi‐element therapy transducer. To ensure accurate delivery of treatment, the accuracy of the assumed bubble cloud location and the registration between CBCT and robotic arm should be tested prior to patient treatment. By having regular quality assurance (QA) tests in place, small deviations in the bubble cloud location can be accounted for or recalibration can be performed if the deviation exceeds a threshold.

QA is an essential part of image‐guided therapy delivery.^15^, ^16^ With an adequate acoustic window, ultrasound‐guided histotripsy can provide real‐time feedback on treatment accuracy by cavitation bubble cloud visualization throughout the treatment duration. A limitation of CBCT‐guided histotripsy is the inability to visualize cavitation in real‐time.^12^ This limitation does not restrict the ability to perform treatment, however, reinforces the need for assurance of targeting accuracy prior to treatment initiation for CBCT‐guided histotripsy to be widely utilized clinically.^17^

Animal tumor models can be utilized to simulate a realistic scenario for a targetable tumor; however, added variables such as acoustic aberration and respiratory motion make it difficult to quantify the targeting error source. Furthermore, the creation of these models is expensive and time‐consuming, and using animal models for ongoing QA would be inappropriate.

Phantoms provide a low‐cost, controlled, and reproducible environment for the development, characterization, and QA of targeting techniques. Phantoms mimicking parenchymal and connective tissue have previously been described for histotripsy treatment parameter characterization and development.^17^, ^18^, ^19^, ^20^, ^21^, ^22^, ^23^, ^24^, ^25^ Of note, Maxwell et al.^18^ proposed an agarose gel‐based phantom with suspended bovine red blood cells for ultrasound evaluation of histotripsy. This phantom allowed clear visualization of histotripsy treatments on ultrasound as hypo‐echoic regions caused by the rupture of individual red blood cells. However, these phantoms could not demonstrate histotripsy treatment zones in CBCT imaging because x‐ray attenuation contrast is not provided by histotripsy treatments in a homogenous medium. Consequently, Kutlu et al.^17^ developed an agarose gel‐based multi‐layered phantom (MLP) with alternating layers of high (barium‐containing) and low (non‐barium‐containing) x‐ray attenuation coefficient. In these MLPs, histotripsy treatment caused adjacent layers in the target volume to mix and created a distinct x‐ray visible treatment zone. MLPs were utilized in CBCT‐targeting development and validation. Strategies to incorporate distinct barium sulfate patterns and distributions in CBCT‐guided histotripsy phantoms have also been investigated to mitigate the long and multi‐step MLP construction process.^26^ However, these methods were shown to provide poor histotripsy treatment zone visualization. Another limitation of the MLP included the lack of a fiducial or target. Therefore, the targeting accuracy was indirectly calculated by the distance of treatment zone borders to phantom edges.^12^, ^17^ A phantom with an embedded treatable target can be used in the direct assessment of targeting accuracy and treatment completeness. A preliminary clinical workflow for CBCT‐guided histotripsy can include regular interval QA treatments of target‐embedded phantoms to ensure adequate calibration prior to patient treatments. In this study, we present an ultrasound and x‐ray compatible imaging phantom with an embedded target for (1) fine‐tuning the assumed bubble cloud location to improve CBCT‐guided histotripsy accuracy, (2) providing a preliminary technique for daily QA, and (3) the development of strategies for margin cleanup in previously incomplete treatments.

## 2 MATERIALS AND METHODS

### 2.1 Phantom construction

#### 2.1.1 Spherical target construction

**FIGURE 1:**
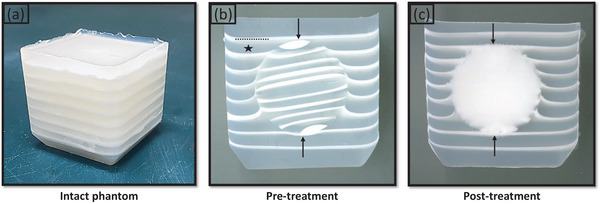
(a) Optical image of an intact cubical multi‐layered phantom. (b) Optical scan of a pre‐treatment phantom central cut‐down demonstrating the embedded spherical target (arrows), barium containing white layers (dashed line), and non‐barium containing translucent layers (star). (c) Optical scan of a post‐treatment phantom central cut‐down demonstrating histotripsy‐induced homogenization of neighboring layers in the embedded target (arrows).

The multi‐layered structure of this phantom was based on work published by Kutlu et al.^17^ Pure agar powder (Thermo Scientific, Waltham, Massachusetts, USA) and deionized water were mixed at room temperature (1.5 g agar per 100 mL water) to prepare the material for lower‐attenuation layers. A higher‐attenuation layer material was prepared by adding barium sulfate to the lower‐attenuation material (3 g barium sulfate per 100 mL water). Beakers containing the two mixtures were stirred and heated until solutions were homogenized at ∼85°C. The solutions were then placed in a vacuum chamber (BVV, Naperville, Illinois, USA) and degassed under increasing negative pressure up to ∼ ‐100 kPa. Following degassing, the beakers were placed on magnetic heated stirrer plates and maintained at ∼60°C (50°C–70°C) while layered targets were created.^27^ Spherical 2.8 cm diameter targets were created by pouring alternating mixture types into hemispherical molds. Syringes were used in the pouring process to control the layer thickness. The layer thicknesses were on the order of a typical cavitation bubble cloud (∼3 × 3 × 6 mm) to ensure adequate visualization of histotripsy‐induced mixing of neighboring layers (Figure 1). Approximately seven alternating layers of ∼1 mm thickness barium and ∼4 mm non‐barium layers were added to each hemisphere. The hemispheres were then joined together using the non‐barium agar solution as an adhesive. Spheres were left to set for 10 min and subsequently stored in degassed deionized water.

#### 2.1.2 Outer phantom construction

**FIGURE 2:**
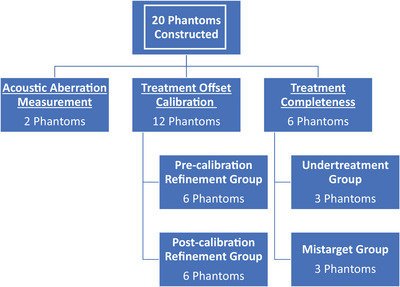
The allocation of manufactured phantoms into different study sections and groups is shown in a workflow chart.

Each spherical target was embedded in a larger multi‐layered phantom (Figure 1). The outer phantoms were constructed in cubical 5 × 5 × 5 cm (*n* = 17) or cylindrical 6.5 × 6.5 × 5 cm molds (*n* = 3). The two types of agar‐based mixtures that form the layers were prepared as described in Methods Section 2.1.1. The bottom four layers [alternating non‐barium (∼4 mm) and barium (∼1 mm) containing] were poured into the molds and, to prevent the target from sinking to the bottom, were left to set for approximately 3 min. Subsequently, the spherical target was placed on top such that its layers were oriented parallel to the outer phantom layers. The parallel orientation was chosen so that the target layers and the outer phantom layers would be perpendicular to the long axis of a histotripsy bubble cloud to allow better mixing. More alternating layers were poured around the spherical target, with care being taken to keep layer thickness consistent and to not have any air bubbles trapped between the target and outer layers. Twenty phantoms were created (3 cylindrical, 17 cubical) and stored in deionized water at 4°C temperature, ∼ ‐100 kPa pressure until they were treated. These phantoms were assigned to and utilized in experimental groups as stated in Figure 2.

### 2.2 Study design

Experiments were conducted to assess the phantom's ability to document offsets between the treatment zone and the target after CBCT‐guided histotripsy.

A research prototype histotripsy system (HistoSonics, Plymouth, Minnesota, USA) was used to deliver all treatments. The prototype had a 700‐kHz, 56‐element concave therapy transducer and a coaxially aligned 3‐MHz curvilinear array ultrasonic imaging probe (5C1e Curved Array; BK Medical, Peabody, Massachusetts, USA). The therapy transducer geometrical focal point was nominally 16 cm from the transducer surface. Bubble cloud cavitation treatment was delivered in short pulses (< 20 µs), with peak negative pressure > 14 MPa, and at a low duty cycle (< 1%).

**FIGURE 3:**
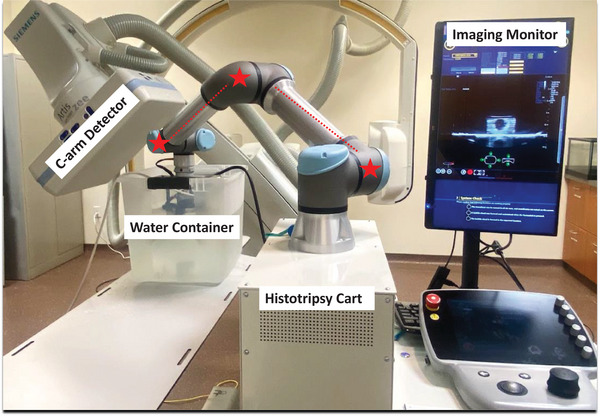
Cone‐beam CT‐guided histotripsy experimental set‐up demonstrating histotripsy cart, robotic arm joints (red dashed lines, stars), water container holding phantom, and x‐ray system C‐arm. The therapy transducer and phantom are in the water container and cannot be directly visualized in this figure.

The histotripsy system robotic arm (Figure 3) was registered to an Artis zee C‐arm CBCT (Siemens Healthineers, Forcheim, Germany) coordinate system in order to automatically target locations selected in a pre‐treatment CBCT.^28^ To this end, the mobile histotripsy cart was positioned alongside the x‐ray table, and care was taken to ensure C‐arm clearance during image acquisition rotations. As described in Wagner et al.,^11^ a helical fiducial phantom was attached to the end effector of the robotic arm, and a hand‐eye calibration step using CBCT and fluoroscopic imaging was performed. After this step, the therapy transducer was attached to the robotic arm end effector, replacing the helical calibration phantom.

**FIGURE 4:**
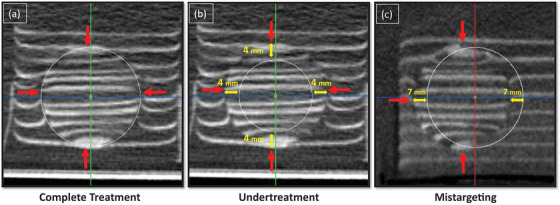
Treatment planning software interface (red, green, and blue solid lines; white circles), with overlayed annotations (red and yellow arrows). Red arrows demonstrate 28 mm spherical target and yellow arrows demonstrate planning differences in undertreatment and mistargeting groups. Planned treatment zones for various experiments are shown (white circle). (a) Planned treatment zones for pre‐ and post‐calibration refinement groups encompass the complete target volume. (b) Undertreatment group's 20 mm treatment zone is planned to centrally treat the target with a 4 mm untreated margin. (c) Mistarget group's 28 mm treatment zone is planned to partially treat the target with a 7 mm lateral offset from the center.

Target‐containing treatable phantoms were fixed to the bottom of a 33 × 44 × 28 cm container filled with deionized degassed water used for acoustic coupling. The water container holding the phantom was placed on the x‐ray table and pre‐treatment CBCT images were acquired (see Figure 3). For treatment planning, custom treatment planning software based on the ImFusion framework (ImFusion GmbH, Munich, Germany)^11^, ^12^ was used to select desired spherical treatment volumes in the pre‐treatment CBCT. The treatment planning software interface can be seen in Figure 4.

The center of the planned treatment volume was ∼2.5 cm from the upper phantom border in each treatment. Spherical treatments were created by the helical movement of the cavitation bubble cloud through the planned target volume by the automated robotic arm joint movement. Each phantom was treated twice with identical transducer head locations and treatment parameters to ensure adequate homogenization of the treatment volume. Post‐treatment CBCTs were acquired in each phantom. The water bath set‐up was not moved between pre‐ and post‐treatment imaging to facilitate analysis of the treatment zone.^18^

#### 2.2.1 Acoustic aberration measurement

Differences in the speed of sound between tissues and the water bath may cause phase shifts, which can result in a change in cavitation bubble cloud location.^29^ The purpose of this sub‐investigation was to assess whether the materials used for the phantom would cause notable changes (aberration) in the bubble cloud location due to the differences in the speed of sound compared to water. Large aberrations would limit the utility of the phantom to predict targeting accuracy since the results would not be representative of the targeting accuracy observed in vivo.

The aberration offset was measured using proprietary software provided by the manufacturer (HistoSonics, Plymouth, Minnesota, USA).^30^ First, a bubble cloud was formed in a water bath, while acquiring live diagnostic ultrasound images with the coaxially mounted imaging transducer. The location of the bubble cloud relative to the diagnostic transducer was manually annotated in the US images. The same procedure was then used to measure the location of the bubble cloud in the phantom. Since both measurements were relative to the diagnostic transducer, the difference between the two bubble cloud locations (in water and in the phantom) represents the aberration offset measured in mm. To determine whether the phantom causes acoustic aberration, test pulses (single cavitation bubble clouds) were performed in two intact phantoms in the top, middle, and bottom layers of the spherical target, and top and bottom layers of the outer phantom [varied depths (between 5 and 40 mm) from the upper phantom border]. The acoustic aberration offsets visualized on ultrasound (if any) were recorded.

#### 2.2.2 Treatment offset calibration

In contrast to the previous section, this sub‐investigation evaluated the offset between the assumed bubble cloud location (the geometrical focal point of the transducer) and the actual bubble cloud location relative to the therapy transducer. Any errors caused by registration of the robotic arm to the CBCT coordinate space would also be apparent in this study.

The histotripsy system was registered to the CBCT system as described in Methods Section ‘Study design’. The offset between the assumed and actual bubble cloud location was determined in six phantoms by measuring the offset between the planned treatment volume and actual treatment volume. To this end, pre‐treatment CBCT images were acquired, and the location was selected such that the planned treatment volume aligned exactly with the 2.8 cm embedded spherical targets as seen in Figure 4a.

Planned treatment locations and diameters were recorded to calculate the offset between planned and actual treatment regions. The actual treatment zones were manually segmented using 3D Slicer (version 5.0.3) in the post‐treatment CBCTs and compared to the planned treatment zones. The treatment centers were calculated as the centroid (first image moment) of the binary mask representing the segmentation. Treatment offsets were calculated as the difference between the centroids of the planned and actual treatment segmentations in the X, Y, and Z directions. Errors > 1 mm in any axis were used to refine the therapy transducer location calibration by adding the offset to the geometrical focal point of the transducer. Treatment volume diameters in X, Y, and Z directions were manually measured on post‐treatment CBCTs using a clinical workstation (Leonardo, Siemens Healthineers, Forchheim, Germany).

Assuming the measured treatment offsets are repeatable, the accuracy of the CBCT‐based targeting approach can be improved by including this offset into the targeting software (calibration refinement). To evaluate whether this approach can be used to improve accuracy, a second experiment was conducted, where the measured average offset was built into the targeting approach. Six additional phantoms were treated, and the offsets were measured as described above. To determine significant changes in treatment accuracy after calibration refinement, unpaired two‐sample *t*‐tests were performed comparing the signed treatment offsets in X, Y, and Z directions before and after calibration refinement.

#### 2.2.3 Treatment completeness study

The purpose of this sub‐investigation was the evaluate the ability of the phantom to depict incomplete treatments of the target. Two scenarios were investigated: 1) a treatment that is too small to cover the full target (“*undertreatment group”*) and 2) a treatment that is offset from the center of the target (“*mistargeting group*”). These studies included the calibration refinement described in Methods Section 2.2.2 to achieve the best possible treatment positioning.

For the “undertreatment” group (*N* = 3), planned treatment was centrally aligned on the 28 mm spherical target, and 20 mm treatments were planned as seen in Figure 4b. For the “mistarget” group (*N* = 3), planned treatment was centrally aligned on the 2.8 cm target, and intentionally shifted 7 mm laterally in the Y direction, and 2.8 cm diameter spherical treatments were planned as seen in Figure 4c.

Centroid locations of three different regions were compared: the planned treatment, the actual treatment, and the intact spherical target segmented from the pre‐treatment CBCTs. The latter two were manually segmented utilizing 3D Slicer (version 5.0.3). The *planned to treatment zone accuracy* was quantified as the centroid distances in X, Y, and Z directions, as described previously. Similarly, the *spherical target to treatment zone distance* was measured. The treatment volume diameters in the undertreatment and mistargeting group phantoms were manually measured in X, Y, and Z directions on the post‐treatment CBCTs using a clinical workstation.

#### 2.2.4 Target visibility analysis

To enable accurate segmentation of the target and treatment in CBCT images, good visibility of the regions is important. To this end, the visibility was measured using the local signal‐difference‐to‐noise ratio (SDNR) metric. Due to the alternating dark and bright layers of the phantom, a conventional SDNR measurement is not representative of the target visibility. Therefore, the signal difference was measured for each point along the target boundary and the absolute value was subsequently averaged.

Pre‐treatment CBCT imaging data from the 18 phantoms used in the “Treatment Offset Calibration” and the “Treatment Completeness Study” sections were further analyzed in this section. The visibility of the spherical target in CBCT images was quantified by measuring the edge strength along the outer surface of the target. To this end, intact spherical targets in pre‐treatment CBCTs were manually segmented utilizing 3D Slicer (version 5.0.3). The surface of the intact target was calculated using the marching cubes algorithm, and normal vectors were determined for all surface points. At each surface point, the local gradient was calculated as the difference between the grayscale intensity values of a pair of corresponding points inside and outside of the target. The points were identified by adding or subtracting the normal vector to the surface point, respectively. Similar to conventional SDNR measurements, the mean absolute gradient over all surface points was then divided by the standard deviation of the noise measured in six layers in each outer phantom (three barium, three non‐barium). The data analysis was performed using Matlab (R2021a, The MathWorks, Inc., Natick, Massachusetts, USA).

#### 2.2.5 Treatment zone visibility analysis

Post‐treatment cone‐beam CT imaging data from the 18 phantoms used in the “Treatment Offset Calibration” and the “Treatment Completeness Study” sections were further analyzed in this section. The visibility of treatment zones in calibration refinement, undertreatment, and mistargeting groups was quantified to characterize the phantom's ability to demonstrate distinct target, outer phantom, and treatment zones. In the calibration refinement groups, SDNRs between histotripsy treatment zones and intact outer phantom non‐barium and barium layers were reported. Mean and standard deviations of the grayscale values inside regions of interest (ROIs) were analyzed based on the work described by Kutlu et al.^17^ Circular ROIs were centrally placed in the coronal, axial, and sagittal planes in the treatment zone. The three treatment zone ROIs had a mean *µ_t_* and a standard deviation σ*_t_*. Similarly, *µ_l_* and σ*_l_* were measured in three ROIs for the barium and non‐barium layers separately. To this end, ROIs were placed in three consecutive barium, and three consecutive non‐barium layers in the outer phantom parallel to the orientation of the layers. The SDNRs were calculated using

(1) $$SDNR\;=\frac{\left|\mu_{t}-\mu_{l}\right|}{\sqrt{{\sigma_{t}}^{2}+{\sigma_{l}}^{2}}}\;$$

**FIGURE 5:**
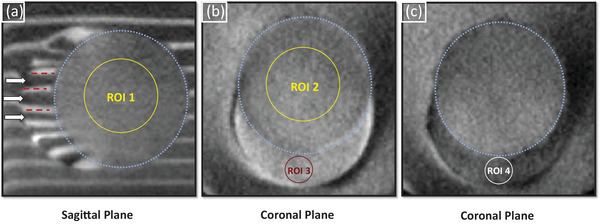
Cross‐sectional cone‐beam CT slices of a mistargeted phantom demonstrating region of interest selection for the signal difference to noise ratio analysis. Treatment volumes are outlined by blue dashed circles. (a) Sagittal plane showing treatment zone ROI 1 and three consecutive barium (red dashes) and non‐barium (white arrows) layers where ROIs were analyzed. (b, c) Coronal slices demonstrating treatment zone, and intact target barium and non‐barium layer ROIs in ROI 2, ROI 3, and ROI 4, respectively.

SDNRs between the treatment zone and intact target layers were analyzed in the undertreatment and mistargeting groups to quantify treatment zone visibility relative to the intact target in incomplete treatment scenarios. To this end, three ROIs were placed in the treatment zone yielding *µ_t_* and σ*_t_*. Three ROIs were placed in consecutive target barium layers, and three ROIs were placed in consecutive target non‐barium layers (see Figure 5) yielding *µ_l_* and σ*_l_*. SDNRs were calculated utilizing the same formula as in the calibration refinement groups.

## 3 RESULTS

All treatments were successfully performed. The total treatment time was approximately 20 min for each 28 mm diameter treatment and 7 min for each 20 mm diameter treatment. Measurement uncertainties in the following sections are given as average ± standard deviation (SD).

### 3.1 Acoustic aberration measurement

Testing two intact phantoms for acoustic aberrations at five different depths revealed no noticeable acoustic aberration at any depth within the phantoms, possibly due to the shallow nature of the treatments. Because all phantoms were treated in a consistent environment, the acoustic aberration correction step was not needed and, therefore, foregone in the following experiments.

### 3.2 Treatment offset calibration

**FIGURE 6:**
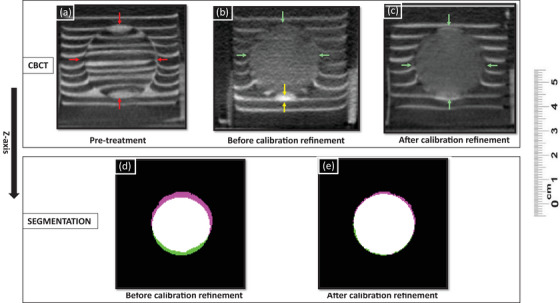
Target‐embedded phantom demonstrates use in improving cone‐beam CT‐guided histotripsy accuracy. (a) Pre‐treatment CBCT utilized for targeting, red arrows depict 2.8 cm target. (b) Post‐treatment before calibration refinement CBCT visually demonstrates a 2 mm calibration error in the Z‐axis (yellow arrows), utilized for calibration fine‐tuning. Green arrows depict the treatment zone. (c) After calibration refinement for the 2 mm offset, phantom demonstrates visually improved targeting accuracy. (d, e) Central axial slice of planned (green) and actual (purple) treatment volume segmentation overlap (perfect overlap = white) demonstrates targeting accuracy improvement in the Z‐axis. The distance between planned and actual treatment zone centroids was less than 1 mm in the post‐calibration refinement segmentation.

The offset between planned and actual treatment volume centroids before calibration refinement was 0.2 ± 0.2, 0.4 ± 0.2, and 2.4 ± 0.2 mm in X, Y, and Z directions (Z being the direction of ultrasound propagation) (Figure 6d). This 2 mm error in Z was corrected when targeting the post‐calibration refinement group.

After refining calibration, offsets between planned and actual treatment volume centroids were 0.2 ± 0.1, 0.2 ± 0.1, and 0.4 ± 0.2 mm in X, Y, and Z directions (Figure 6e). The Student's *t*‐test analysis showed a statistically significant (*p* < 0.001) difference in treatment offset in the Z direction before (mean = 2.4 mm, SD = 0.2 mm, *n* = 6) and after calibration refinement (mean = 0.4 mm, SD = 0.2 mm, *n* = 6). No statistically significant differences were observed in X and Y directions. Individual centroid offset measurements for each phantom in this section are provided in Table 1.

**TABLE 1:** Treatment offset measurements.

	X‐offset in mm	Y‐offset in mm	Z‐offset in mm
Measured phantom offsets before calibration refinement (BCR)			
Phantom BCR1	0.55	0.38	**‐2.11**
Phantom BCR2	0.30	0.19	**‐2.19**
Phantom BCR3	0.15	0.10	**‐2.22**
Phantom BCR4	0.09	0.55	**‐2.55**
Phantom BCR5	0.08	0.56	**‐2.50**
Phantom BCR6	‐0.16	0.40	**‐2.68**
**Mean ± standard dev**.	**0.17 ± 0.24**	**0.36 ± 0.19**	**‐2.38 ± 0.23**
Measured phantom offsets after calibration refinement (ACR)			
Phantom ACR1	0.16	‐0.28	**‐0.51**
Phantom ACR2	0.25	‐0.30	**‐0.55**
Phantom ACR3	0.18	‐0.31	**‐0.29**
Phantom ACR4	0.15	‐0.18	**‐0.34**
Phantom ACR5	0.30	‐0.16	**0.04**
Phantom ACR6	0.35	‐0.10	**‐0.63**
Mean ± standard dev.	**0.23 ± 0.08**	** −0.22 ± 0.09**	** −0.38 ± 0.24**

*Note*: Individual measured offsets (mm) in the X, Y, and Z directions are listed for each phantom before and after calibration refinement.

Values of the Z‐offset (column) are demonstrated in bold caracters.

The planned treatment zone was a 28 mm diameter sphere, and the measured treatment zone dimensions were 28.2 ± 0.5, 28.1 ± 0.6, and 28.5 ± 0.5 mm in the X, Y, and Z directions for the pre‐calibration refinement group and 27.8 ± 0.4, 28.4 ± 0.6, and 28.2 ± 0.3 mm for the post‐calibration refinement group.

In the estimation of the offset, the average difference seen from the planned and actual treatment volume centroid (as measured by manual segmentation) was used. The six individual differences were all within 1 mm of each other in the X, Y, and Z directions, which indicates that no single measurement contributed to the estimation of the offset more than any other measurement.

### 3.3 Treatment completeness

The 2 mm calibration error measured from the calibration refinement group was accounted for in the histotripsy transducer focal point to CBCT coordinates system registration step for all subsequent treatments.

**FIGURE 7:**
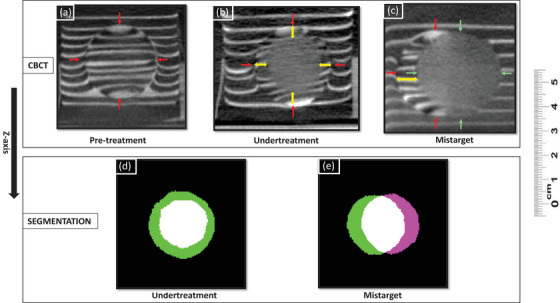
Target‐embedded phantom demonstrates use in assessing the accuracy and completeness of treatments. (a) Pre‐treatment cone‐beam CT, utilized for targeting. Red arrows demonstrate 2.8 cm target, treatments were planned to achieve incomplete treatments of the spherical target. (b) Phantom depicts residual untreated target in undertreatment. Yellow arrows depict the 4 mm untreated target margin, the 2 cm treatment is located centrally. (c) Phantom depicts residual untreated target in a mistargeting scenario. Green arrows demonstrate the treatment zone, and the yellow arrow demonstrates a 7 mm lateral offset. (d, e) A central slice of intact target and treatment volume segmentation overlap (white volume) demonstrates (d) central undertreatment and (e) 7 mm lateral mistargeting. Non‐overlapping target and treatment zones are seen in green and purple, respectively.

In the undertreatment group, the distance between planned and actual treatment zone centroids was 0.5 ± 0.5, 0.2 ± 0.1, and 0.6 ± 0.2 mm in X, Y, and Z directions. Similarly, the spherical target to treatment zone distance was 0.4 ± 0.1, 0.6 ± 0.1, and 0.2 ± 0.1 mm in X, Y, and Z directions. The planned treatment zone was a 20 mm diameter sphere, and the measured treatment zone dimensions were 19.5 ± 0.4, 19.9 ± 0.5, and 20.1 ± 1.2 mm in X, Y, and Z directions (see Figure 7b, d).

In the mistarget group, the planned to treatment zone accuracy was 0.2 ± 0.3, 0.2 ± 0.1, and 0.1 ± 0.2 mm in X, Y, and Z directions. The treatment zone was planned to have a 7 mm lateral (Y direction) offset relative to the spherical target, and the spherical target to treatment zone distance was 0.4 ± 0.3, 6.8 ± 0.2, and 0.2 ± 0.1 mm in X, Y, and Z directions (see Figure 7c, e). The planned treatment zone was a 28 mm diameter sphere, and the measured treatment zone dimensions for the mistarget group were 28.2 ± 0.5, 28.4 ± 0.4, and 28.5 ± 0.8 mm.

### 3.4 Target and treatment zone visibility

Target edge visibility strength (comparable to a conventional SDNR) was 4.2 ± 0.6, and all targets were segmented in the planning stage.

Treatment zone SDNR relative to the outer phantom layers in the pre‐ and post‐calibration refinement groups was 1.6 ± 1.0 and 2.9 ± 0.8 for barium and non‐barium layers, respectively. In the undertreatment group, treatment zone SDNR relative to the intact spherical target was 1.3 ± 0.3 and 2.2 ± 0.1 for barium and non‐barium layers, respectively. In the mistarget group, treatment zone SDNR relative to the intact target was 2.4 ± 0.4 and 1.6 ± 0.7 for barium and non‐barium layers, respectively.

## 4 DISCUSSION

The proposed phantom allowed cavitation location calibration and improved targeting accuracy. Intact target, intact outer phantom, and treatment zones were all visually discernable in pre‐calibration refinement, mistargeting, and undertreatment scenarios. A small error of 2 mm was detected before calibration refinement and submillimeter accuracy was achieved in X, Y, and Z directions in post‐calibration refinement group phantoms. When the treatment was intentionally shifted 7 mm relative to the target, the treatment zone accurately reflected the shift. This phantom with an embedded target can be used to fine‐tune the calibration, assess completeness of treatments, and facilitate development of strategies for treating previously incompletely treated targets. Potential applications also include periodic QA and end‐to‐end evaluation of targeting accuracy.

The spherical targets were easily visualized in all CBCTs, and edge strength assessment quantified pre‐treatment target visibility. The measured edge visibility metric is conceptually equivalent to SDNR measurements but accounts for the phantom's layered structure. The measured edge strength shows that the signal difference at the target's boundary is on average four times higher than the noise. The visible edge of the target was formed by misalignment between the target and outer phantom layers at the target interface, and the slight curvature of the target edges due to the nature of the hemispherical molds used in phantom construction. This visible edge allowed target segmentation for treatment planning and assessment. Histotripsy disrupts the layered pattern of an intact phantom, resulting in a homogenized treatment zone distinct from the untreated phantom. Thus, this phantom allows visualization, and therefore segmentation, of the distinct residual target, intact outer layers, and treatment zones in incorrect calibration or incomplete treatment scenarios.

In this study, centroid location analysis resulted in a < 1 mm standard deviation within all groups of treated phantoms. Therefore, a CBCT‐targeted histotripsy treatment in one phantom can provide sufficient information to finetune the therapy transducer location to correct any existing calibration errors prior to treating patients. Furthermore, the embedded spherical target allowed direct visualization and measurement of targeting errors on post‐treatment CBCT without the need for sophisticated analysis, which makes this phantom ideal for QA.

A limitation of this study includes manual segmentation of references the offset measurements were made on. As manual segmentation can introduce errors, automated segmentation of phantom imaging can be developed through future research to mitigate such errors. Furthermore, the manual phantom creation process is labor intensive and creating a batch (5–10) of phantoms from scratch can take up to 5 hours. Automated fabrication can facilitate widespread clinical and laboratory use. An automated approach can also mitigate potential variations in the thickness of individual layers in the phantom caused by the manual pouring and cooling process. New strategies can be developed to create targetable phantoms with higher sensitivity to very small treatments and individual bubble clouds.

## 5 CONCLUSION

The target containing phantom allowed treatment location calibration to improve targeting accuracy, according to manually segmented references. The phantom also accurately depicted the planned treatment zone size. When the treatment was intentionally shifted relative to the target, the visualized treatment zone reflected this shift with submillimeter accuracy. This investigation suggests this prototype phantom is suitable for preliminary QA of CBCT‐guided histotripsy.

## AUTHOR CONTRIBUTIONS

All co‐authors have contributed to writing and reviewing this manuscript and have approved the submitted version. Furthermore, all co‐authors agree to be accountable for all aspects of the work in ensuring that questions related to the accuracy or integrity of any part of our work are appropriately investigated and resolved. Authors Kutlu, Wagner, Laeseke, and Speidel were involved in the experimental design and phantom design. Authors Kutlu, Minesinger, and Wagner were responsible for the acquisition, analysis, or interpretation of experimental data.

## CONFLICT OF INTEREST STATEMENT

Dr. Wagner is a consultant for HistoSonics. Dr. Laeseke is a consultant for HistoSonics, NeuWave (Johnson & Johnson/Ethicon), and Elucent Medical and a sharedholder of HistoSonics, Elucent Medical, McGinley Orthopedics, and RevOps Health.
